# Supplementary material for: First-Principles Study on Mechanical and Optical Behavior of Plutonium Oxide under Typical Structural Phases and Vacancy Defects
Source: Materials (Basel). 2022 Nov 4;15(21):7785. doi: 10.3390/ma15217785 (PMC9656257; doi:10.3390/ma15217785)
Supplement: Supplementary file 1 [file materials-15-07785-s001.zip › materials-1996006-supplementary.pdf]

Supporting Information

First-principles study of mechanical and optical behavior of plutonium oxide under typical structural phases and vacancy defects

Jin-Xing Cheng [1,*], Fei Yang [2], Qing-Bo Wang [1], Yuan-Yuan He [2], Yi-Nuo Liu [2], Zi-Yu Hu [2,*], Wei-Wei Wen [1], You-Peng Wu [1], Cheng-Yin Zheng [1], Ai Yu [1], Xin Lu [1], Yue Zhang [1]

[1] Beijing Institute of high technology, Beijing 100094, China;
[2] College of Mathematics and Physics, Beijing University of Chemical Technology, Beijing 100029, China
* Correspondence: chengjx@tsinghua.org.cn (J.-X.C.); huziyu@mail.buct.edu.cn (Z.-Y.H.)

Table S1. Energies of AFM, FM and NM for β-Pu_2O_3, α-Pu_2O_3, γ-Pu_2O_3, PuO, α-PuO_2, γ-PuO_2.

Plutonium oxide	AFM energy(eV)	FM energy(eV)	NM energy(eV)
β-Pu_2O_3	-57.565	-57.803	-53.971
α-Pu_2O_3	-114.427	-115.147	-106.712
γ-Pu_2O_3	-56.971	-57.476	-53.661
PuO	-95.555	-95.326	-88.435
α-PuO_2	-133.580	-134.194	-122.877
γ-PuO_2	-66.202	-66.560	-66.373

Crystal Coordinate Lists:

1. β-Pu_2O_3

```
Beta-Pu2 O3
  1.00000000000000
    3.8821335015818668    -0.0000000000000000    -0.0000000000000000
   -1.9410667507909334     3.3620262332915520     0.0000000000000000
   -0.0000000000000000    -0.0000000000000000     5.8473959514856864
  Pu    O
  2     3
 Direct
 0.3333333429999996  0.6666666870000029  0.2392150939910920
 0.6666666870000029  0.3333333429999996  0.7607849200089091
 0.0000000000000000  0.0000000000000000  0.0000000000000000
 0.3333333429999996  0.6666666870000029  0.6469546544250036
 0.6666666870000029  0.3333333429999996  0.3530453155749937
```

2. α-Pu_2O_3

```
Alpha-Pu4 O6
  1.00000000000000
     5.4591667217853903        0.0000000000000000       -0.0000000000000000
    -0.0000000000000000        5.4591667217853903       -0.0000000000000000
     0.0000000000000000        0.0000000000000000        5.4591667217853903
  Pu      O
  4       6
Direct
 0.7500000000000000    0.7500000000000000    0.2500000000000000
 0.2500000000000000    0.7500000000000000    0.7500000000000000
 0.7500000000000000    0.2500000000000000    0.7500000000000000
 0.2500000000000000    0.2500000000000000    0.2500000000000000
 0.5000000000000000    0.0000000000000000    0.0000000000000000
-0.0000000000000000   -0.0000000000000000    0.5000000000000000
 0.5000000000000000    0.0000000000000000    0.5000000000000000
 0.0000000000000000    0.5000000000000000    0.5000000000000000
-0.0000000000000000    0.5000000000000000    0.0000000000000000
 0.5000000000000000    0.5000000000000000    0.0000000000000000
```

3. γ-Pu_2O_3

```
Gamma-Pu2 O3
  1.00000000000000
     3.8128837783244780        0.0000000000000000        0.0000000000000000
     0.0000000000000000        3.8128837783244780        0.0000000000000000
     0.0000000000000000        0.0000000000000000        5.4622533667408222
  Pu      O
   2      3
Direct
 0.5000000000000000   -0.0000000000000000    0.7656124900952080
-0.0000000000000000    0.5000000000000000    0.2343874799047897
-0.0000000000000000    0.0000000000000000    0.0000000000000000
 0.5000000000000000    0.5000000000000000   -0.0000000000000000
-0.0000000000000000    0.0000000000000000    0.5000000000000000
```

4. PuO

```
Pu4 O4
  1.00000000000000
     5.1000370814982325        0.0000000000000000       -0.0000000000000000
    -0.0000000000000000        5.1000370814982325       -0.0000000000000000
     0.0000000000000000       -0.0000000000000000        5.1000370814982325
  Pu      O
  4       4
Direct
 0.5000000000000000    0.0000000000000000   -0.0000000000000000
```

0.5000000000000000 0.5000000000000000 0.5000000000000000
-0.0000000000000000 -0.0000000000000000 0.5000000000000000
-0.0000000000000000 0.5000000000000000 0.0000000000000000
0.0000000000000000 0.0000000000000000 0.0000000000000000
0.0000000000000000 0.5000000000000000 0.5000000000000000
0.5000000000000000 0.0000000000000000 0.5000000000000000
0.5000000000000000 0.5000000000000000 0.0000000000000000

5. *α*-PuO₂

```
Alpha-Pu4 O8
  1.00000000000000
     5.4423906392051418     0.0000000000000000    -0.0000000000000000
     0.0000000000000000     5.4423906392051418     0.0000000000000000
    -0.0000000000000000     0.0000000000000000     5.4423906392051418
  Pu    O
  4     8
Direct
0.0000000000000000  -0.0000000000000000  -0.0000000000000000
-0.0000000000000000  0.5000000000000000  0.5000000000000000
0.5000000000000000  0.0000000000000000  0.5000000000000000
0.5000000000000000  0.5000000000000000  -0.0000000000000000
0.2500000000000000  0.2500000000000000  0.7500000000000000
0.2500000000000000  0.7500000000000000  0.7500000000000000
0.2500000000000000  0.7500000000000000  0.2500000000000000
0.2500000000000000  0.2500000000000000  0.2500000000000000
0.7500000000000000  0.2500000000000000  0.2500000000000000
0.7500000000000000  0.7500000000000000  0.2500000000000000
0.7500000000000000  0.7500000000000000  0.7500000000000000
0.7500000000000000  0.2500000000000000  0.7500000000000000
```

6. *γ*-PuO₂

```
Gamma-Pu2 O4
  1.00000000000000
     5.0696852698140988     0.0000000000000000     0.0000000000000000
     0.0000000000000000     5.0696852698140988     0.0000000000000000
     0.0000000000000000     0.0000000000000000     3.5456764326349082
  Pu    O
   2    4
Direct
0.5000000000000000  0.5000000000000000 -0.0000000000000000
0.0000000000000000  0.0000000000000000  0.5000000000000000
0.8106049711684941  0.8106049711684941 -0.0000000000000000
0.1893950138315082  0.1893950138315082  0.0000000000000000
0.3106050011684966  0.6893950288315059  0.5000000000000000
0.6893950288315059  0.3106050011684966  0.5000000000000000
```

7. β-$Pu_{16}O_{23}$

```
Beta-Pu16 O23
  1.00000000000000
     7.1758626404266215    0.0000000451123670   -0.0000000000000000
    -3.5879321086188645    6.2144788854106379   -0.0000000000000000
    -0.0000000000000001    0.0000000000000000   11.6250200270611277
  Pu    O
  16    23
Direct
0.1666666719999981  0.3333333429999996  0.1181211672423131
0.1666666719999981  0.3333333429999996  0.6172712263064412
0.1666302434863120  0.8333151587419565  0.1182710862193360
0.1646512803791248  0.8323256772102655  0.6184605288179018
0.6666849012580485  0.3333697715136858  0.1182710862193360
0.6676743827897395  0.3353487346208656  0.6184605288179018
0.6666849012556434  0.8333151587443616  0.1182710862193360
0.6676743828311326  0.8323256771688726  0.6184605288179018
0.3333223971021258  0.1666776178978717  0.3825402788742450
0.3359068749331593  0.1640931400668384  0.8771727175439581
0.3333223970975540  0.6666447951996833  0.3825402788742450
0.3359068749238237  0.6718137508569866  0.8771727175439581
0.8333552648003215  0.1666776179024439  0.3825402788742450
0.8281863091430184  0.1640931400761741  0.8771727175439581
0.8333333730000021  0.6666666870000029  0.3904430732544931
0.8333333730000021  0.6666666870000029  0.9017610773262582
0.0068830695353564  0.0137661390686800  0.9993116966997139
0.0000296294369735  0.0000592588795829  0.5024912400280551
0.0068830695333237  0.4931169304666764  0.9993116966997139
0.0000296294426090  0.4999703705573910  0.5024912400280551
0.5000000000000000  0.0000000000000000  -0.0054983588374144
0.5000000000000000  0.0000000000000000  0.5056616817751977
0.4862338609313200  0.4931169304646436  0.9993116966997139
0.4999407411204174  0.4999703705630265  0.5024912400280551
0.1666666719999981  0.3333333429999996  0.3183736671736397
0.1666666719999981  0.3333333429999996  0.8170230838173305
0.1650763838033789  0.8325382289029001  0.3192498879029815
0.1369721414523165  0.8184861077130765  0.8092954057361971
0.6674618310971049  0.3349236311966187  0.3192498879029815
0.6815139522869285  0.3630278735476872  0.8092954057361971
0.6674618310995124  0.8325382289004924  0.3192498879029815
0.6815139522607664  0.8184861077392399  0.8092954057361971
0.3344489205743142  0.1655510944256834  0.1830976282071985
0.3341954303921517  0.1658045846078459  0.6815372557674932
0.3344489205740486  0.6688978421483734  0.1830976282071985
```

0.3341954303996595   0.6683908617918151   0.6815372557674932
0.8311022178516314   0.1655510944259490   0.1830976282071985
0.8316091982081898   0.1658045846003381   0.6815372557674932
0.8333333730000021   0.6666666870000029   0.1833116605504539

8. ***β*-Pu16O22**

Beta-Pu16 O22
1.00000000000000
7.1260304100758693   0.0000000512925963   -0.0000000000000000
-3.5630159823449219   6.1713229144091688   0.0000000000000001
0.0000000000000000   0.0000000000000001   11.6232405387481847
Pu   O
16   22
Direct
0.1666666719999981   0.3333333429999996   0.1176294735541783
0.1666666719999981   0.3333333429999996   0.6176294665541814
0.1627943617019736   0.8313972178690093   0.1189146884978388
0.1627943617019736   0.8313972178690093   0.6189146814978415
0.6686028421309956   0.3372056532980173   0.1189146884978388
0.6686028421309956   0.3372056532980173   0.6189146814978415
0.6686028421670347   0.8313972178329775   0.1189146884978388
0.6686028421670347   0.8313972178329775   0.6189146814978415
0.3364093043696105   0.1635907106303882   0.3786246707916261
0.3364093043696105   0.1635907106303882   0.8786246707916261
0.3364093043463470   0.6728186097159600   0.3786246707916261
0.3364093043463470   0.6728186097159600   0.8786246707916261
0.8271814502840439   0.1635907106536508   0.3786246707916261
0.8271814502840439   0.1635907106536508   0.8786246707916261
0.8333333730000021   0.6666666870000029   0.4101755083050965
0.8333333730000021   0.6666666870000029   0.9101755083050966
0.0074492805972532   0.0148985612038494   0.0014168149243278
0.0074492805972532   0.0148985612038494   0.5014168149243279
0.0074492806065965   0.4925507193934041   0.0014168149243278
0.0074492806065965   0.4925507193934041   0.5014168149243279
0.5000000000000000   -0.0000000000000000   -0.0002798694351671
0.5000000000000000   -0.0000000000000000   0.4997201305648327
0.4851014387961500   0.4925507194027468   0.0014168149243278
0.4851014387961500   0.4925507194027468   0.5014168149243279
0.1666666719999981   0.3333333429999996   0.3176738927273645
0.1666666719999981   0.3333333429999996   0.8176738927273647
0.1338445745558428   0.8169223242736049   0.3102336294183179
0.1338445745558428   0.8169223242736049   0.8102336294183180
0.6830777357264001   0.3661554404441519   0.3102336294183179
0.6830777357264001   0.3661554404441519   0.8102336294183180
0.6830777357177608   0.8169223242822470   0.3102336294183179

0.6830777357177608   0.8169223242822470   0.8102336294183180
0.3355129891933669   0.1644870258066310   0.1826610183173968
0.3355129891933669   0.1644870258066310   0.6826610333173946
0.3355129891977238   0.6710259793910868   0.1826610183173968
0.3355129891977238   0.6710259793910868   0.6826610333173946
0.8289740806089181   0.1644870258022741   0.1826610183173968
0.8289740806089181   0.1644870258022741   0.6826610333173946

9. *β*-$Pu_{16}O_{20}$

```
Beta-Pu16 O20
  1.00000000000000
     7.0098730056175818    -0.0064061483531669     0.0036648264263567
    -3.5104851977894831     6.0803369881608234    -0.0000000005576083
     0.0047771540781246     0.0027580912295320    11.6819367286318965
  Pu    O
  16    20
Direct
0.1592354559608737    0.3296177349943026    0.1195273080919397
0.1592354559608737    0.3296177349943026    0.6195273010919428
0.1592354559608737    0.8296177649943055    0.1195273080919397
0.1592354559608737    0.8296177649943055    0.6195273010919428
0.6793526270973417    0.3396763130346965    0.1193231020061895
0.6793526270973417    0.3396763130346965    0.6193230950061926
0.6793526270973417    0.8396763430346986    0.1193231020061895
0.6793526270973417    0.8396763430346986    0.6193230950061926
0.3348905643978078    0.1674452826931959    0.3757348427631041
0.3348905643978078    0.1674452826931959    0.8757348427631041
0.3348905643978078    0.6674452976932007    0.3757348427631041
0.3348905643978078    0.6674452976932007    0.8757348427631041
0.8224783437697910    0.1612391573907815    0.4073485186548398
0.8224783437697910    0.1612391573907815    0.9073485186548399
0.8224783437697910    0.6612391723907862    0.4073485186548398
0.8224783437697910    0.6612391723907862    0.9073485186548399
0.0168841567211484    0.0084420783561872    0.0012576240548242
0.0168841567211484    0.0084420783561872    0.5012576240548240
0.0168841567211484    0.5084420783561870    0.0012576240548242
0.0168841567211484    0.5084420783561870    0.5012576240548240
0.4764100516626502    -0.0117949741639153    0.0006215469848563
0.4764100516626502    -0.0117949741639153    0.5006215469848567
0.4764100516626502    0.4882050258360844    0.0006215469848563
0.4764100516626502    0.4882050258360844    0.5006215469848567
0.1323573518245924    0.3161786829194566    0.3102353032603719
0.1323573518245924    0.3161786829194565    0.8102353032603720
0.1323573518245924    0.8161787129194592    0.3102353032603719
0.1323573518245924    0.8161787129194592    0.8102353032603720
```

```
0.7036736750183923    0.3518368369994740    0.3045168434729929
0.7036736750183923    0.3518368369994740    0.8045168434729931
0.7036736750183923    0.8518368669994766    0.3045168434729929
0.7036736750183923    0.8518368669994766    0.8045168434729931
0.3413845505474065    0.1706922757758139    0.1821863977108768
0.3413845505474065    0.1706922757758139    0.6821864127108745
0.3413845505474065    0.6706922907758187    0.1821863977108768
0.3413845505474065    0.6706922907758187    0.6821864127108745
```

10. β-$Pu_{16}O_{18}$

```
Beta-Pu16 O18
  1.00000000000000
     6.9172239034526770        0.0000000787835836        0.0000000000000000
    -3.4586126811472209        5.9904912028527422       -0.0000000000000000
    -0.0000000000000000       -0.0000000000000000       11.7021015138445410
  Pu     O
  16     18
Direct
0.1626335323346142    0.3373664826653839    0.1192754192901326
0.1626335323346142    0.3373664826653839    0.6192754122901358
0.1626335323517062    0.8252670936863259    0.1192754192901326
0.1626335323517062    0.8252670936863259    0.6192754122901358
0.6747329663136785    0.3373664826482913    0.1192754192901326
0.6747329663136785    0.3373664826482913    0.6192754122901358
0.6666666870000029    0.8333333730000021    0.1184415931183013
0.6666666870000029    0.8333333730000021    0.6184415861183044
0.3333333429999996    0.1666666719999981    0.3741997627496674
0.3333333429999996    0.1666666719999981    0.8741997627496675
0.3390540844468474    0.6695270577170092    0.4009582948104927
0.3390540844468474    0.6695270577170092    0.9009582948104927
0.8304730022829958    0.1609459305531510    0.4009582948104927
0.8304730022829958    0.1609459305531510    0.9009582948104927
0.8304730022701603    0.6695270577298440    0.4009582948104927
0.8304730022701603    0.6695270577298440    0.9009582948104927
0.0162931029689371    0.0081465514782358    0.0030792537590917
0.0162931029689371    0.0081465514782358    0.5030792537590917
-0.0000000000000000   0.5000000000000000    0.0011729506557776
-0.0000000000000000   0.5000000000000000    0.5011729506557777
0.4918534485092917    0.0081465514907071    0.0030792537590917
0.4918534485092917    0.0081465514907071    0.5030792537590917
0.4918534485217641    0.4837068970310640    0.0030792537590917
0.4918534485217641    0.4837068970310640    0.5030792537590917
0.1497205337047119    0.3502794812952837    0.3046181899391343
0.1497205337047119    0.3502794812952837    0.8046181899391341
0.1497205337217261    0.7994410964264461    0.3046181899391343
```

0.1497205337217261	0.7994410964264461	0.8046181899391341
0.7005589635735608	0.3502794812782715	0.3046181899391343
0.7005589635735608	0.3502794812782715	0.8046181899391341
0.6666666870000029	0.8333333730000021	0.3004030491210535
0.6666666870000029	0.8333333730000021	0.8004030491210534
0.3333333429999996	0.1666666719999981	0.1842436479586338
0.3333333429999996	0.1666666719999981	0.6842436629586315
